# Revisiting the Role of First Trimester Homocysteine as an Index of Maternal and Fetal Outcome

**DOI:** 10.1155/2014/123024

**Published:** 2014-05-05

**Authors:** Mariano Mascarenhas, Syed Habeebullah, M. G. Sridhar

**Affiliations:** ^1^Department of Reproductive Medicine, Christian Medical College, Vellore 632004, India; ^2^Department of Obstetrics and Gynecology, Jawaharlal Institute of Postgraduate Medical Education and Research (JIPMER), Pondicherry 605005, India; ^3^Department of Biochemistry, Jawaharlal Institute of Postgraduate Medical Education and Research (JIPMER), Pondicherry 605005, India

## Abstract

*Aim.* To revisit the role of first trimester homocysteine levels with the maternal and fetal outcome. *Methods.* This was a cohort study comprising 100 antenatal women between 8 and 12 weeks of gestation. Serum homocysteine levels were checked after overnight fasting. *Results.* There were significantly elevated homocysteine levels among women with prior history of hypertensive disorders of pregnancy and prior second or third trimester pregnancy losses. There was no significant difference in homocysteine levels among women with previous gestational diabetes mellitus, preterm deliveries, or fetal malformations. Homocysteine levels were significantly elevated in those who developed hypertensive disorder of pregnancy, oligohydramnios, and meconium stained amniotic fluid, had a pregnancy loss, or delivered a low birth weight baby. There was no significant difference in homocysteine levels for those who developed gestational diabetes mellitus. *Conclusions.* Increased first trimester serum homocysteine is associated with history of pregnancy losses, hypertensive disorders of pregnancy, and preterm birth. This is also associated with hypertensive disorders of pregnancy, pregnancy loss, oligohydramnios, meconium stained amniotic fluid, and low birth weight in the current pregnancy. This trial is registered with ClinicalTrials.gov CTRI/2013/02/003441.

## 1. Introduction

Homocysteine is an amino acid which has sprung into prominence in the past few decades [1]. Homocysteine is intricately linked to folate metabolism and one methyl transfer. Elevated homocysteine levels have been shown to be deleterious on vascular endothelium [1, 2]. Elevated homocysteine has also served as an early marker for insulin resistance due to the effects of insulin on homocysteine metabolism and renal clearance [3]. These relationships of homocysteine to disease states in the nonpregnant adult population have been extrapolated to link it to the pregnancy specific conditions of gestational diabetes mellitus and hypertensive disorders of pregnancy. Homocysteine levels decline during pregnancy [4], and the levels are the lowest during second trimester of pregnancy and increase in the second half of the third trimester of pregnancy. Hence, samples taken within strict time frame, such as 4 weeks (between 8 to 12 weeks of gestation), would have a better success at correlating the homocysteine levels with the pregnancy outcome, by minimising the gestational age bound variation of homocysteine. In addition, most maternal complications such as hypertensive disorders of pregnancy and gestational diabetes mellitus develop much later, so timing the sample collection before 12 weeks would sufficiently predate the onset of complications.

Serum homocysteine levels in pregnancy have been linked to preeclampsia [4–6], recurrent abortions [7–9], and low birth weight [10]. But evidence on this is conflicting with some studies stating that serum homocysteine values have no correlation to maternal and fetal outcome [11]. Hence, there is a need for this study to shed light on these conflicting opinions regarding the effect of homocysteine levels on both maternal and fetal outcomes.

## 2. Aim and Objectives

To correlate the levels of homocysteine in late first trimester of pregnancy (8–12 weeks) with the maternal and fetal outcome of pregnancy, especially with regard to development of hypertensive disorders of pregnancy and gestational diabetes mellitus.

To estimate the homocysteine levels in late 1st trimester (8–12 weeks) and to correlate it with prior history of (i) first, second, and third trimester pregnancy losses, (ii) hypertensive disorders of pregnancy and gestational diabetes mellitus, (iii) fetal malformations, and (iv) preterm delivery and birth weight.

To correlate the homocysteine levels with the present pregnancy—parameters, namely, (i) hypertensive disorder of pregnancy, (ii) gestational diabetes mellitus, (iii) pregnancy loss, (iv) amniotic fluid volume and meconium staining of amniotic fluid, and (v) birth weight.

## 3. Materials and Methods

It was a prospective observational cohort study comprising antenatal women who attended the antenatal outpatient department of a tertiary care—university level health centre. The study was started after obtaining the Institutional Ethics Committee approval and was conducted from August 2009 to July 2011. 110 women were invited to participate in the study and 100 agreed to participate in the study. Ten women refused to participate in the study as they were from distant places from our institute and hence could not come in the early morning in fasting state for the homocysteine test. Subjects were enrolled at 8 to 12 weeks gestation and known diabetics or hypertensives on treatment were excluded. At enrollment, a questionnaire was collected regarding age, period of gestation, parity, abortions with respective period of gestation and whether induced or spontaneous, history of previous fetal malformations, history of hypertensive disorders of pregnancy or gestational diabetes mellitus in previous pregnancies, and birth weight of previous children and whether the previous deliveries were term or preterm births. Venous blood samples were taken after overnight fasting for serum homocysteine estimation and serum separated by centrifugation and sera assayed for homocysteine by chemiluminescence assay technique using a well-calibrated fully automated chemiluminescence assay system—the Advia Centaur CPTM immunoassay system (Siemens, Germany) which is an automated, random access—direct chemiluminescent immunoassay analyzer [12]. The patients were followed up in antenatal OPD as per hospital protocol. They were asked for symptoms of preeclampsia and GDM. Routine examination included blood pressure recordings, pedal edema, urine albumin and sugar, Symphysio Fundal Height, and clinically amniotic fluid level status. Glucose challenge test was done at 32 weeks of pregnancy, and if abnormal, 100 gram oral glucose tolerance test (OGTT) was done to diagnose Gestational Diabetes Mellitus. Antenatal Ultrasound was done if there was clinical suspicion of IUGR (e.g., Symphysio Fundal Height less than 4 cm than corresponding to period of gestation), and in case of malpresentations, hypertensive disorders of pregnancy (and other risk factors for IUGR) and before induction of labour. Doppler ultrasound of umbilical and middle cerebral artery was done in case of IUGR and preeclampsia to do assess the risk of fetal morbidity in these cases.

Statistical analysis was done using GraphPad Instat software; using unpaired *t*-test for comparison of two parametrically distributed groups of data, unpaired *t*-test with Welch correction was used for comparison of two parametrically distributed groups of data with unequal variances. Mann-Whitney *U* test was used for comparison of two nonparametrically distributed groups of data, and one way ANOVA was used for more than two parametrically distributed groups of data. Kruskal-Wallis test was used for comparison of more than two parametrically distributed groups of data. *P* value of ≤0.05 was taken as significant and ≤0.001 was taken as highly significant.

## 4. Results

Of the 100 antenatal women recruited, 10 were lost to follow up thus leaving 90 subjects whose outcomes were analysed. The ages of the subjects were in the range of 21 to 33 years with a mean age of 24.76 ± 2.6 years. The BMI of the subjects ranged from 15.5 kg/m^2^ to 33.2 kg/m^2^ and the mean was 21.70 ± 6.23 kg/m^2^. The mean serum homocysteine level was 14.07 ± 7.84 *μ*mol/L. There was no correlation between the homocysteine levels and the age, BMI, the period of gestation, or the number of folic acid tablets taken by the subjects.

Of the recruited 100 women, 14 women had past history of hypertensive disorders of pregnancy, 5 women had previous gestational diabetes mellitus, 6 had a previous preterm delivery, and 2 had a previous fetal malformation. There was significantly elevated homocysteine levels (*P* = 0.0359) among women with prior history of hypertensive disorders of pregnancy. However, the difference in homocysteine levels between those with and without past history of gestational diabetes mellitus was not statistically different (*P* = 0.054). There was no statistically significant difference of homocysteine levels with history of prior preterm deliveries (*P* = 0.8348) or with history of previous fetal malformation (*P* = 0.078) (Table 1).

Of the 100 antenatal women, 27 women had previous one and 9 women had previous two first trimester pregnancy losses, 9 women had previous one and 3 had previous two second trimester pregnancy losses, and 9 women had previous one third trimester pregnancy loss. There was no statistically significant elevation of homocysteine levels in those with history of prior first trimester pregnancy losses (*P* = 0.1687). There was statistically significant elevation of homocysteine levels in those women with history of prior second trimester pregnancy losses (*P* = 0.0307). There was highly significant elevation of homocysteine levels in those women with history of prior third trimester pregnancy losses (*P* < 0.0001). Comparing women with no history of prior pregnancy losses in any trimester and those with pregnancy losses in any trimester, there was highly significant elevation of homocysteine levels in the latter group (Table 2). There was no significant correlation between prior birth weights and the serum homocysteine levels (Table 2).

The present pregnancy outcome of the 90 women who completed follow-up (10 women were lost to follow up) was then analysed. Of the 90 women, 18 developed hypertensive disorders of pregnancy (HDP) and 7 developed gestational diabetes mellitus (GDM); 2 patients had a fetus with a congenital malformation and 8 patients had a pregnancy loss (including 2 stillbirths). Of the 84 antenatal women whose delivery outcomes were analysed: 18 women had oligohydramnios, 17 women had meconium stained amniotic fluid, and 11 women delivered low birth weight babies (birth weight < 2500 gm).

The homocysteine levels were statistically elevated in those who went on to develop HDP than those who did not develop HDP (*P* = 0.0011). The power of the study to detect a difference in HDP was 0.95. There was no significant difference (*P* = 0.6312) in homocysteine levels between those who developed and those who did not develop GDM. The power of the study to detect a difference in GDM was 0.42 (Table 3).

There was significant elevation in homocysteine levels (*P* = 0.0002) among those who suffered a pregnancy loss when compared to those who had a live birth in the current pregnancy. There was no statistically significant difference in homocysteine levels (*P* = 0.6621) between those who had a fetus with a congenital malformation and those who had a morphologically normal fetus. There was statistically significant increase in homocysteine levels in women with oligohydramnios on comparing with women with normal amniotic fluid levels (*P* < 0.001), and women with meconium stained amniotic fluid had higher homocysteine levels than those with clear amniotic fluid (*P* = 0.0037). The serum homocysteine levels were significantly elevated (*P* = 0.0224) for those women who gave birth to low birth weight babies than those whose babies weighed above 2500 grams at birth (Table 4).

## 5. Discussion

Serum homocysteine is not significantly correlated with the age of the subjects, which is in agreement with another Indian study by Das et al. [13]. Serum homocysteine levels are not significantly associated with body mass index, in concordance with a study by Han et al. [14]. There was no significant variation with the mean period of gestation at which the samples were taken with the serum homocysteine concentrations. This may be due to the factor that the time window in which the samples were taken itself was short, and the study by Murphy et al. [15] and Dodds et al. [4] on the homocysteine levels longitudinally through pregnancy suggests that the homocysteine levels may be more stabilised from 8 weeks of gestation onward up to the middle of second trimester; there is a reduction in homocysteine levels prior to 8 weeks and a rise in homocysteine levels in the third trimester.

There was no significant correlation between homocysteine levels and the number of folic acid tablets taken in correlation with the prior studies [4, 16]. The difference in homocysteine levels in those with prior first trimester losses did not reach statistical significance in this study.

However, when all prior pregnancy losses were taken together, the difference in homocysteine levels was statistically significant; the same statistical significance held true with those who had prior second or third trimester losses. This can be compared to other studies, which have also showed that prior early pregnancy losses [8, 9, 17] and stillbirths [18] are reflected in higher homocysteine levels. Those with prior hypertensive disorders of pregnancy also had increase in homocysteine levels, in concordance with other studies [18, 19].

Prior preterm birth was not significantly associated with homocysteine levels, in contrast to a study by Kramer et al. [2] which saw an association of preterm birth with higher homocysteine levels, but the proposed mechanism of decidual vasculopathy of the placenta due to higher homocysteine level could not be proven by that study. The serum homocysteine levels was not different between those who had a previous fetal malformation in contrast with the Hordaland Homocysteine study [18] which showed an increased incidence of malformations, particularly neural tube defects, in those mothers with elevated homocysteine levels. There was a declining trend of prior birth weights with higher homocysteine levels confirmed by other studies [18]. With reference to the index pregnancy, the serum homocysteine levels were significantly different between those who went on to develop hypertension during their pregnancy, and those who remained normotensive, in conformance with prior studies [5–7, 20].* Post priori* power calculation has shown that the power to detect a difference in hypertensive disorder of pregnancy was 0.95 which indicates that the present study was adequately powered to show that elevated homocysteine is associated with development of hypertensive disorders of pregnancy.

The serum homocysteine levels were not significantly different between those who developed gestational diabetes mellitus and those who maintained normal glucose tolerance, similar to a study by Idzior-Waluś et al. [21]. However, it is in contrast to a study by Tarim et al. [22] who found a significantly elevated homocysteine level in antenatal women with gestational diabetes mellitus. Nevertheless, much conclusion cannot be drawn from the present study as the* post priori* power calculated was 0.42 only. The serum homocysteine levels were not different between those who had fetuses with congenital malformations and normal fetuses, in conformance with the study [23] of Wang et al. Elevated serum homocysteine levels were also associated with pregnancy loss in the current pregnancy. This is in concordance with several other studies which also showed an increase in the rate of spontaneous abortions [8] and stillbirths [18, 24] in pregnancies with elevated homocysteine levels.

The difference in homocysteine levels between those with oligohydramnios and normal amniotic fluid level status and those with clear and meconium stained amniotic fluid has been shown to be statistically significant in this study. However, there is a lack of previous studies to show this association. However, this can be taken as an extrapolation of the higher incidence of preeclampsia [5–7, 20, 25], fetal growth restriction [26], and impairment of placental transport [27] that has been documented in previous studies. This can also be taken as an extrapolation of the increased incidence of hypertensive disorders of pregnancy and its associated placental insufficiency. The difference in serum homocysteine levels between those with low birth weight babies and those whose birth weight was above 2500 grams was statistically significant. This is in conformance with most studies [26, 28] but one study [5] contradicted stating that there was no statistical difference.

## 6. Limitations

The* a priori* power calculation in the study was based on prior study on serum homocysteine levels in hypertensive disorders of pregnancy. Due to paucity of data on serum homocysteine levels in gestational diabetes mellitus,* a priori* power calculation for gestational diabetes was not done. The* post priori* power calculation was 0.42 which indicates that this study may not have had the power to detect a difference in homocysteine levels in gestational diabetes. The number of cases was small and definite conclusions cannot be derived from this study. However, this study is the first of its kind in this ethnic population in a developing country. Further studies will be needed to strengthen these conclusions. Though the number of folic acid tablets taken prior to the sample collection for homocysteine is quantified (by memory recall of the patients), the number of folic acid tablets taken by the patients after the sample collection during the rest of the pregnancy may be a confounding factor, especially due to the fact that though folic acid is routinely recommended during first trimester, the focus shifts to iron during the second and third trimester, notwithstanding the fact that many iron preparations contain folic acid in addition to iron. Vitamin B12 levels were not estimated and this is a confounder as well. Serum folate and vitamin B12 values were not assayed in the samples. This is a limitation of this study since homocysteine values vary with folate and B12 levels.

## 7. Conclusions

Serum homocysteine levels in late first trimester (8 to 12 weeks) of pregnancy are significantly associated with prior pregnancy losses, particularly in the second and third trimesters and with prior hypertensive disorders of pregnancy. With reference to the current pregnancy, increased serum homocysteine levels are also significantly associated with hypertensive disorders of pregnancy, pregnancy loss, oligohydramnios, meconium stained amniotic fluid, and low birth weight. However, raised homocysteine levels are not significantly associated with body mass index or with gestational diabetes mellitus and fetal malformations, neither in the past pregnancies nor in the current pregnancy.

## Figures and Tables

**Table 1:** Homocysteine levels with reference to outcome of past pregnancies.

	No past history of hypertensive disorder of pregnancy (*n* = 86)	Past history of hypertensive disorder of pregnancy (*n* = 14)	*P* value	Statistical test used
Serum homocysteine (*μ*mol/L)	13.11 ± 5.81	19.13 ± 9.34	0.04*	Unpaired *t*-test—Welch correction

	No prior gdm (*n* = 95)	Prior gdm (*n* = 5)		

Serum homocysteine (*μ*mol/L)	13.83 ± 6.82	20.39 ± 7.05	0.054	Unpaired *t*-test

	No prior preterm delivery (*n* = 94)	Prior preterm delivery (*n* = 6)		

Serum homocysteine (*μ*mol/L)	14.33 ± 7.20	13.76 ± 4.20	0.83	Mann-Whitney *U* test

	No previous fetal malformation (*n* = 98)	Previous fetal malformation (*n* = 2)		

Serum homocysteine (*μ*mol/L)	14.01 ± 6.80	23.88 ± 9.12	0.08	Mann-Whitney *U* test

*P ≤ 0.05 significant.

**Table 2:** Homocysteine levels with reference to outcome of past pregnancies.

	No prior first trimester loss (*n* = 64)	One prior first trimester loss (*n* = 27)	Two prior first trimester losses (*n* = 9)		*P* value	Test used
Serum homocysteine (*μ*mol/L)	12.80 ± 6.58	15.25 ± 7.58	17.08 ± 5.80		0.1687	One way ANOVA

	No prior second trimester loss (*n* = 88)	One prior second trimester loss (*n* = 9)	Two prior second trimester losses (*n* = 3)			

Serum homocysteine (*μ*mol/L)	13.59 ± 7.02	16.72 ± 2.89	6.52 ± 0.50		0.0307*	Kruskal-Wallis test

	No intrauterine fetal death in the third trimester (*n* = 91)	One previous intrauterine fetal death in the third trimester (*n* = 9)				

Serum homocysteine (*μ*mol/L)	13.16 ± 5.75	23.79 ± 9.21			<0.0001**	Unpaired *t*-test

	No prior pregnancy loss (any trimester) (*n* = 52)	One prior pregnancy loss (*n* = 25)	Two prior pregnancy losses (*n* = 19)	Three prior pregnancy losses (*n* = 4)		

Serum homocysteine (*μ*mol/L)	11.08 ± 4.18	13.46 ± 7.15	19.23 ± 6.65	17.80 ± 6.41	0.0002**	Kruskal-Wallis test

*P ≤ 0.05 significant.

**P ≤ 0.001 highly significant.

**Table 3:** Homocysteine levels with reference to maternal outcome of present pregnancy.

	Normotensive in present pregnancy (*n* = 72)	Hypertensive disorders in present pregnancy (*n* = 18)	*P* value	Statistical test used
Serum homocysteine (*μ*mol/L)	13.51 ± 7.95	18.44 ± 6.59	0.0011*	Mann-Whitney *U* test

	No gestational diabetes in present pregnancy (*n* = 83)	Gestational diabetes in present pregnancy (*n* = 7)		

Serum homocysteine (*μ*mol/L)	14.41 ± 7.98	15.66 ± 7.61	0.6312	Mann-Whitney *U* test

*P ≤ 0.05 significant.

**Table 4:** Homocysteine levels with reference to fetal outcome of present pregnancy.

	No fetal congenital malformation (*n* = 88)	Fetal congenital malformation (*n* = 2)	*P* value	Statistical test used
Serum homocysteine (*μ*mol/L)	14.56 ± 8.0	12.07 ± 1.75	0.6621	Mann-Whitney *U* test

	No pregnancy loss (*n* = 82)	Pregnancy loss (*n* = 8)		

Serum homocysteine (*μ*mol/L)	13.51 ± 7.47	24.65 ± 4.51	0.0002**	Mann-Whitney *U* test

	Clear amniotic fluid (*n* = 67)	Meconium stained amniotic fluid (*n* = 17)		

Serum homocysteine (*μ*mol/L)	12.52 ± 6.94	18.43 ± 8.48	0.0037*	Mann-Whitney *U* test

	No oligohydramnios (*n* = 66)	Oligohydramnios (*n* = 18)		

Serum homocysteine (*μ*mol/L)	12.58 ± 7.01	20.02 ± 7.92	<0.0001**	Mann-Whitney *U* test

	Birth weight ≥2500 gm (*n* = 73)	Birth weight <2500 gm (*n* = 11)		

Serum homocysteine (*μ*mol/L)	12.35 ± 5.64	23.22 ± 12.32	0.0224*	Unpaired *t*-test—Welch correction

*P ≤ 0.05 significant.

**P ≤ 0.001 highly significant.

## References

[1] Forges T, Monnier-Barbarino P, Alberto JM, Guéant-Rodriguez RM, Daval JL, Guéant JL (2007). Impact of folate and homocysteine metabolism on human reproductive health. *Human Reproduction Update*.

[2] Kramer MS, Kahn SR, Rozen R (2009). Vasculopathic and thrombophilic risk factors for spontaneous preterm birth. *International Journal of Epidemiology*.

[3] Meigs JB, Jacques PF, Selhub J (2001). Fasting plasma homocysteine levels in the insulin resistance syndrome. *Diabetes Care*.

[4] Dodds L, Fell DB, Dooley KC (2008). Effect of homocysteine concentration in early pregnancy on gestational hypertensive disorders and other pregnancy outcomes. *Clinical Chemistry*.

[5] Cotter AM, Molloy AM, Scott JM, Daly SF (2001). Elevated plasma homocysteine in early pregnancy: a risk factor for the development of severe preeclampsia. *American Journal of Obstetrics and Gynecology*.

[6] Cotter AM, Molloy AM, Scott JM, Daly SF (2003). Elevated plasma homocysteine in early pregnancy: a risk factor for the development of nonsevere preeclampsia. *American Journal of Obstetrics and Gynecology*.

[7] Holmes VA, Wallace JMW, Alexander HD (2005). Homocysteine is lower in the third trimester of pregnancy in women with enhanced from continued folic acid supplementation. *Clinical Chemistry*.

[8] Yin Y, Zhang T, Dai Y, Zheng X, Pei L, Lu X (2009). Pilot study of association of anembryonic pregnancy with 55 elements in the urine, and serum level of folate, homocysteine and S-adenosylhomocysteine in Shanxi Province, China. *Journal of the American College of Nutrition*.

[9] Guillén MR, Sánchez LT, Chen J (2009). Dietary consumption of B vitamins, maternal MTHFR polymorphisms and risk for spontaneous abortion. *Salud Pública de México*.

[10] Yajnik CS, Deshpande SS, Jackson AA (2008). Vitamin B12 and folate concentrations during pregnancy and insulin resistance in the offspring: the Pune Maternal Nutrition Study. *Diabetologia*.

[11] Hogg BB, Tamura T, Johnston KE, DuBard MB, Goldenberg RL (2000). Second-trimester plasma homocysteine levels and pregnancy-induced hypertension, preeclampsia, and intrauterine growth restriction. *American Journal of Obstetrics and Gynecology*.

[12] (2005). *Homocysteine Analysis By ADVIA Centaur Automated System*.

[13] Das M, Ghose M, Borah NC, Choudhury N (2010). A community based study of the relationship between homocysteine and some of the life style factors. *Indian Journal of Clinical Biochemistry*.

[14] Han YS, Ha EH, Park HS, Kim YJ, Lee SS (2011). Relationships between pregnancy outcomes, biochemical markers and pre-pregnancy body mass index. *International Journal of Obesity*.

[15] Murphy MM, Scott JM, McPartlin JM, Fernandez-Ballart JD (2002). The pregnancy-related decrease in fasting plasma homocysteine is not explained by folic acid supplementation, hemodilution, or a decrease in albumin in a longitudinal study. *American Journal of Clinical Nutrition*.

[16] Wallace JMW, Bonham MP, Strain JJ (2008). Homocysteine concentration, related B vitamins, and betaine in pregnant women recruited to the Seychelles Child Development Study. *American Journal of Clinical Nutrition*.

[17] Uva MD, Micco PD, Strina I (2007). Hyperhomocysteinemia in women with unexplained sterility or recurrent early pregnancy loss from Southern Italy: a preliminary report. *Thrombosis Journal*.

[18] Refsum H, Nurk E, Smith AD (2006). The Hordaland Homocysteine Study: a community-based study of homocysteine, its determinants, and associations with disease. *The Journal of Nutrition*.

[19] Girouard J, Giguère Y, Moutquin J-M, Forest J-C (2007). Previous hypertensive disease of pregnancy is associated with alterations of markers of insulin resistance. *Hypertension*.

[20] Ingec M, Borekci B, Kadanali S (2005). Elevated plasma homocysteine concentrations in severe preeclampsia and eclampsia. *Tohoku Journal of Experimental Medicine*.

[21] Idzior-Waluś B, Cyganek K, Sztefko K (2008). Total plasma homocysteine correlates in women with gestational diabetes. *Archives of Gynecology and Obstetrics*.

[22] Tarim E, Yigit F, Kilicdag E (2006). Early onset of subclinical atherosclerosis in women with gestational diabetes mellitus. *Ultrasound in Obstetrics and Gynecology*.

[23] Wang L, Wang F, Guan J (2010). Relation between hypomethylation of long interspersed nucleotide elements and risk of neural tube defects. *American Journal of Clinical Nutrition*.

[24] Ananth CV, Elsasser DA, Kinzler WL (2007). Polymorphisms in methionine synthase reductase and betaine-homocysteine S-methyltransferase genes: risk of placental abruption. *Molecular Genetics and Metabolism*.

[25] Mignini LE, Latthe PM, Villar J, Kilby MD, Carroli G, Khan KS (2005). Mapping the theories of preeclampsia: the role of homocysteine. *Journal of Obstetrics and Gynecology*.

[26] Murphy MM, Scott JM, Arija V, Molloy AM, Fernandez-Ballart JD (2004). Maternal homocysteine before conception and throughout pregnancy predicts fetal homocysteine and birth weight. *Clinical Chemistry*.

[27] Jansson T (2009). Novel mechanism causing restricted fetal growth: does maternal homocysteine impair placental amino acid transport?. *Journal of Physiology*.

[28] Fryer AA, Emes RD, Ismail KMK (2011). Quantitative, high-resolution epigenetic profling of CpG loci identifies associations with cord blood plasma homocysteine and birth weight in humans. *Epigenetics*.

